# Neuroprotective effect of paricalcitol in a rat model of transient global cerebral ischemia

**DOI:** 10.1186/s12245-020-00289-7

**Published:** 2020-06-10

**Authors:** Sung Wook Kim, Joo Suk Oh, Jungtaek Park, Hyun Ho Jeong, Young Min Oh, Semin Choi, Kyoung Ho Choi

**Affiliations:** 1 Department of Emergency Medicine, Eunpyeong St. Mary’s Hospital, College of Medicine, The Catholic University of Korea, 1021 Tongil-Ro, Eunpyeong-gu, Seoul, 03312 Republic of Korea; 2 Department of Emergency Medicine, Uijeongbu St. Mary’s Hospital, College of Medicine, The Catholic University of Korea, 271, Cheonbo-Ro, Uijeongbu-si, Gyeonggi-do, 11765 Republic of Korea

**Keywords:** Brain ischemia, Vitamin D, Paricalcitol, Neuroprotection

## Abstract

**Background:**

Paricalcitol is known to attenuate ischemic-reperfusion injury of various organs. However, it is not known whether paricalcitol prevents neuronal injury after global cerebral ischemia. The purpose of this study is to investigate the neuroprotective effect of paricalcitol in a rat model of transient global cerebral ischemia.

**Methods:**

This is a prospective, randomized experimental study. Male Sprague-Dawley rats that survived 10 min of four-vessel occlusion were randomly assigned to two treatment groups: one group was treated with paricalcitol 1 μg/kg IP, and the other was given an equivalent volume of normal saline IP. Drugs were administered at 5 min, 1 day, 2 days, and 3 days after ischemia. Neurologic function was assessed at 2 h, 1 day, 2 days, 3 days, and 4 days after ischemia. We tested motor function 3 days after ischemia using the rotarod test. Also, we tested memory function 4 days after ischemia using the passive avoidance test. We assessed neuronal degeneration in the hippocampus of surviving rats 4 days after ischemia.

**Results:**

Eight rats were allocated to each group. No significant differences were found between the groups in terms of survival rate, motor coordination, or memory function. The neurological function score 2-h post-ischemia was significantly higher in the paricalcitol group (*p* = 0.04). Neuronal degeneration was significantly less in the paricalcitol group compared with the control group (*p* = 0.01).

**Conclusions:**

Paricalcitol significantly attenuated neuronal injury in the hippocampus. Although motor coordination, memory function, and survival rate were not significantly improved by paricalcitol treatment in this study, paricalcitol remains a potential neuroprotective drug after global cerebral ischemia.

## Background

Hypoxic-ischemic brain injuries (HIBI) such as ischemic stroke and post-cardiac arrest syndrome are a leading cause of mortality and disability. Many clinical trials of neuroprotective agents against HIBI have shown disappointing outcomes [1–5], and no effective drugs are currently available. Therefore, further studies are needed to identify potential agents for neuroprotection. Vitamin D exhibits various cellular effects in multiple organs. Vitamin D plays a key role in the regulation of calcium-phosphorus homeostasis. Furthermore, vitamin D3 and 1,25-dihydroxyvitamin D3 (calcitriol), which is an active form of vitamin D3, are well-known immunomodulators with protective effects on inflammatory processes [6–8]. Vitamin D3 has been reported to attenuate ischemia-reperfusion injuries in hepatic and cardiac ischemia [9, 10]. A recent study showed that calcitriol reduced HIBI in a rat model of transient middle cerebral artery occlusion via attenuation of oxidative stress and apoptosis [11, 12]. Moreover, insufficiency of 25-hydroxyvitamin D (calcifediol), which is a prehormone of calcitriol, is known to be related to acute strokes, and low calcifediol levels are a predictor of fatal strokes [13]. 19-nor-1,25-hydroxyvitamin D2 (paricalcitol), which is an active form of vitamin D2, is a synthetic vitamin D2 analog. Paricalcitol is a selective vitamin D receptor activator used for the treatment of secondary hyperparathyroidism. Paricalcitol selectively acts on the parathyroid gland, resulting in a reduced risk of hypercalcemia and hyperphosphatemia. Recently, a few investigators have shown that paricalcitol is protective against renal, cardiac, and hepatic ischemic-reperfusion injury in experimental models [14–17]. Like vitamin D, paricalcitol has significant immunomodulatory activity via vitamin D receptor activation as well as anti-inflammatory and anti-oxidative properties [16].

Collectively, clinical and basic research studies suggest that vitamin D and its synthetic analogs such as paricalcitol play a protective role in ischemic-reperfusion injuries of various organs. Nevertheless, whether paricalcitol prevents HIBI after global cerebral ischemia such as in cases of post-cardiac arrest syndrome is unknown. The purpose of this study is to examine the neuroprotective effect of paricalcitol in a rat model of transient global cerebral ischemia.

## Methods

### Animal preparation

This is a prospective, randomized experimental study. Male Sprague-Dawley rats weighing between 250 and 280 g were provided food and water ad libitum and held at a temperature of 22 °C ± 1 °C under a 12-h light/dark cycle for 5 days prior to the experiments. Transient global cerebral ischemia was induced via the four-vessel occlusion method described in our prior research [18, 19]. To inhibit secretion, atropine (0.01 mg/kg) was administered intraperitoneally before surgery. This was followed by a combination of tiletamine hydrochloride, zolazepam hydrochloride (Zoletil; Virbac, Carros, France) (30 mg/kg), and xylazine (Rompun; Bayer, Monheim, Germany) (10 mg/kg) 10 min later intra-abdominally to induce anesthesia. Thereafter, tracheal intubation was performed to maintain the airway, and the rats were fixed in a prone position in a stereotactic frame. A thermometer probe was inserted into the rectum of the fixed rats, and an automatic temperature control blanket (homeothermic blanket system, NP50-7053-r; Harvard Apparatus, Holliston, MA) was used to maintain normothermia (37 °C ± 0.5 °C) during surgery.

We shaved and sterilized the areas around the cervical regions while the rat was fixed in the prone position and made a 3-cm median incision along the centerline from the border of the lower part of the occipital bone to the back and cervical region. The muscles of the first cervical vertebra were dissected to expose the bilateral alar foramen. A thin needle-shaped electrocautery (SurgiStatTM II; Covidien, Boulder, CO) was inserted approximately 1 to 1.5 mm through the alar foramen, and the bilateral vertebral arteries were permanently occluded by cauterization. Next, the rat was put in a supine position, and the ventral cervical region was shaved and sterilized. A 3-cm median incision was made, and the bilateral common carotid arteries were dissected. Polyethylene tubes (PE-10; BD, Franklin Lakes, NJ) were loosely wrapped around the dissected common carotid arteries to allow both ends of the tubes to emerge approximately 3 cm from the skin, which was then sutured.

**Fig. 1:**
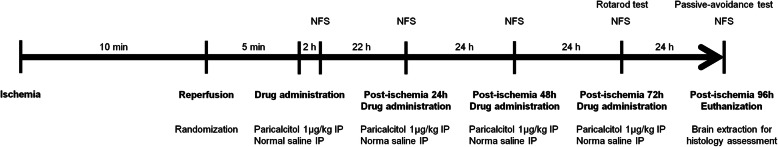
Timeline of experiment. NFS, neurologic function score; IP, intraperitoneal

After surgery, the rats were kept isolated in individual cages and left to recover for 24 h in the same environment as before the surgery. On the following day, the rats were restrained without anesthesia, and the polyethylene tubes were pulled to expose the common artery outside the skin. The common carotid arteries were occluded with microvascular clamps (RS-5422; Roboz, Chicago, IL) for 10 min, and global cerebral ischemia was confirmed by loss of the righting reflex. A thermometer probe was inserted into the rectum to monitor core temperature, and normothermia was maintained using a temperature control blanket throughout the ischemic period. The clamps and polyethylene tubes in the rats that survived global cerebral ischemia were removed 10 min later, and normothermia was maintained until recovery of the righting reflex. We excluded rats in which the righting reflex was maintained during the ischemic period, because this indicated incomplete global cerebral ischemia. Also, we rejected rats without any available outcome variables because of death during the ischemic period or early death within 2-h post-ischemia (Fig. 1).

### Study protocol

The surviving rats were randomly assigned to one of two treatment groups: the paricalcitol group (*n* = 8), which was injected intraperitoneally with paricalcitol (Zemplar; Abbott Laboratories, Abbott Park, IL) (1 μg/kg), and the normal saline group (*n* = 8), which was injected intraperitoneally with an equivalent volume of normal saline. A previous study showed that a low dose of calcitriol (1 μg/kg) reduced HIBI in a rat model of transient middle cerebral artery occlusion [12]. Therefore, we chose to use the low dose of 1 μg/kg to avoid potential side effects such as hypercalcemia and hyperphosphatemia. After recovery of the righting reflex, rats were returned to their cages and observed until 4 days after cerebral ischemia. During the observational period, we injected paricalcitol (1 μg/kg) intraperitoneally, or an equivalent volume of normal saline in the control group, on days 1, 2, and 3 post-ischemia.

### Neurological outcomes

A researcher who was blinded to the treatment measured and recorded the neurological function score (NFS) at 2-h post-ischemia, and then on days 1, 2, 3, and 4 post-ischemia, as previously described [20]. The test consists of five categories representing the level of consciousness, respiration, cranial nerves, motor and sensory function, and coordination. The score ranges from 0 (worst) to 500 (normal) (Table 1).

**Table 1:** Neurological function scoring in rats

Parameter	Characteristic	Score range
General		
Consciousness	Unresponsive, depressed, normal	0, 50, 100
Respiration	Abnormal, normal (60-120)	0, 100
Cranial Nerves		
Olfactory	Orient to smell	No = 0, yes = 20
Vision	Visual stimulus startle response	No = 0, yes = 20
Corneal reflex	Blink response to corneal stimulus	No = 0, yes = 20
Whisker movement	Spontaneous	No = 0, yes = 20
Hearing	Startle response to loud noise	No = 0, yes = 20
Motor		
Left forepaw	Spontaneous or withdrawal from pain	No = 0, yes = 10
Right forepaw	Spontaneous or withdrawal from pain	No = 0, yes = 10
Left hindpaw	Spontaneous or withdrawal from pain	No = 0, yes = 10
Right hindpaw	Spontaneous or withdrawal from pain	No = 0, yes = 10
Tail	Spontaneous or withdrawal from pain	No = 0, yes = 10
Sensory		
Left forepaw	Reaction to pain	No = 0, yes = 10
Right forepaw	Reaction to pain	No = 0, yes = 10
Left hindpaw	Reaction to pain	No = 0, yes = 10
Right hindpaw	Reaction to pain	No = 0, yes = 10
Tail	Reaction to pain	No = 0, yes = 10
Coordination		
Ledge traverse		No = 0, yes = 25
Righting reflex		No = 0, yes = 25
Placing test		No = 0, yes = 25
Stop to table edge		No = 0, yes = 25
Total score		500

### Rotarod test

We assessed motor coordination using the accelerating rotarod test (Model 7750; Ugo Basile, Comerio, Varese, Italy). Four training sessions were performed 5, 4, and 3 days and 1 h prior to ischemic insult. We placed the rats on the stationary rod. After a while, the rod started to rotate at 2 rpm, and it accelerated to 40 rpm within 300 s. If the rats fell off within 5 s after the rod rotated, the trial was repeated. We recorded the latency to fall from the rotating rod. Rats that did not fall off within 300 s were given the maximum score of 300. We obtained a baseline score by averaging the two best scores out of the four training sessions. We performed the rotarod test 3 days after ischemic insult and determined the post-ischemic latency to fall.

### Passive avoidance test

The passive avoidance apparatus (Model 7552; Ugo Basile, Comerio, Varese, Italy) consisted of two sections: the start and escape compartments. The start compartment was illuminated and surrounded by white walls, while the escape compartment was dark, with black walls. The two compartments were connected by an automatic sliding door. Three days after ischemic insult, the rats were exposed to the passive avoidance apparatus 60 min before the acquisition trial. During pre-exposure, the rats were allowed to explore the start compartment for 1 min without access to the escape compartment. Thereafter, the sliding door was opened; the door closed automatically as the rat entered the escape compartment. The rats were then allowed to explore the escape compartment for an additional 1 min. One hour after pre-exposure, the rat was again placed in the start compartment for the acquisition trial, and 10 s later, the door was opened. The latency to step through the door was recorded as baseline retention latency. After the rat entered the escape compartment, the door was closed, and an electrical current (0.8 mA, 2 s) was delivered through the grid floor. The next day, we performed the retention trial. The rat was placed in the start compartment, and 10 s later, the door was opened. The retention latency to enter the escape compartment was recorded. No shock was delivered during the retention trial. If the rat failed to enter the escape compartment within 300 s, it was removed from the apparatus, and the maximum latency of 300 s was recorded. The data was expressed relative to baseline retention latency and used for data analysis.

### Histopathological analysis

After the acquisition trial, tiletamine hydrochloride + zolazepam hydrochloride (1:1 solution, 30 mg/kg) was injected into the abdominal cavity for anesthesia, and the rats were euthanized with 4% paraformaldehyde via transcardiac perfusion fixation. Brains were then post-fixed in 4% paraformaldehyde for more than a day, followed by washing under running water for another day. Finally, they were fixed in paraffin, and two 4 μm-thick coronal sections of the hippocampal Cornu Ammonis 1 (CA1) region from each rat were obtained for hematoxylin-eosin (HE) staining. As each specimen contained right and left CA1 regions, four histological images of a 1.13-mm-long stratum pyramidale were acquired with an optical microscope (IX71; Olympus, Tokyo, Japan). A blinded researcher calculated the percentage of degenerated pyramidal cells in each image using the image analysis software (Image-Pro Premier; Media Cybernetics, Rockville, MD). The median value of the 4 images was calculated for each rat.

### Statistics

Based on the results of our pilot study, we hypothesized that the percentage of injured neurons in the paricalcitol group would be 9 ± 4.5%, while the percentage of injured neurons in the normal saline group would be 30 ± 15%. Assuming a two-sided *α* of 0.05 at a power of 0.8, the necessary number of rats per group was determined to be 8 to reject the null hypothesis. Continuous data are expressed as medians with interquartile ranges (IQR). We conducted Mann-Whitney tests to compare data between the groups. The log-rank test was used to compare the survival distribution between groups. Survival is presented with Kaplan-Meier curves. *P* values less than 0.05 were considered statistically significant.

## Results

### Neurological function and survival

Out of a total of 29 rats subjected to four-vessel occlusion, 23 survived. Of those, four rats that maintained the righting reflex during the ischemic period and three rats that died within 2 h after ischemia were excluded.

**Fig. 2:**
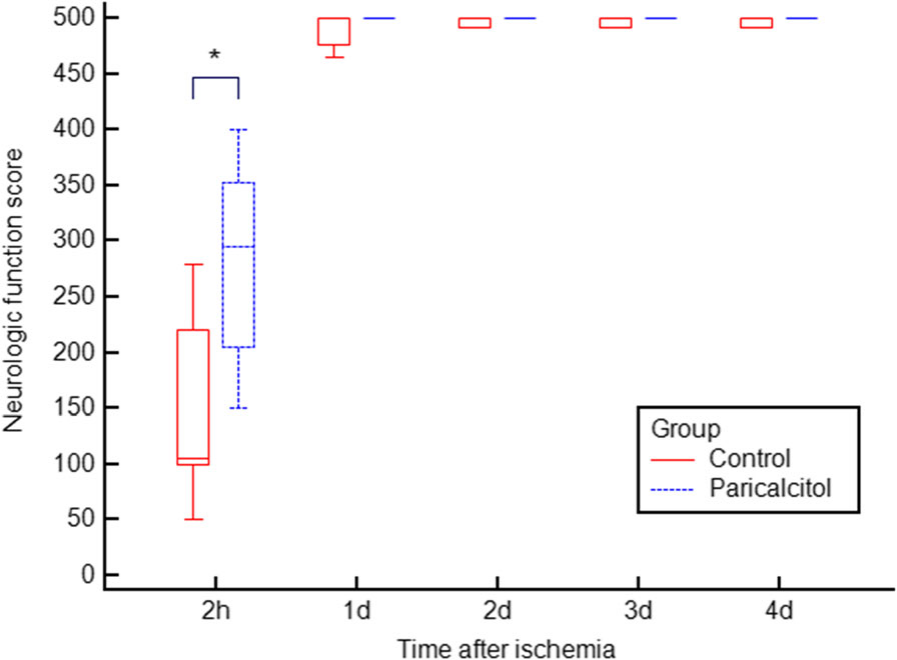
Impact of paricalcitol on neurologic function. Paricalcitol-treated rats showed significantly improved neurologic function at 2 h after cerebral ischemia. Neurologic function scores later than 1 day after ischemia did not demonstrate statistically significant differences between groups (*P = 0.04). The box-and-whisker plot represents medians and interquartile ranges

Paricalcitol treatment significantly improved the 2-h NFS compared with control saline treatment, 295 (IQR, 205 to 352.5) versus 105 (IQR, 100 to 220) (*P* = 0.04). However, the NFSs on days 1, 2, 3, and 4 post-ischemia were not significantly different between the groups (Fig. 2).

**Fig. 3:**
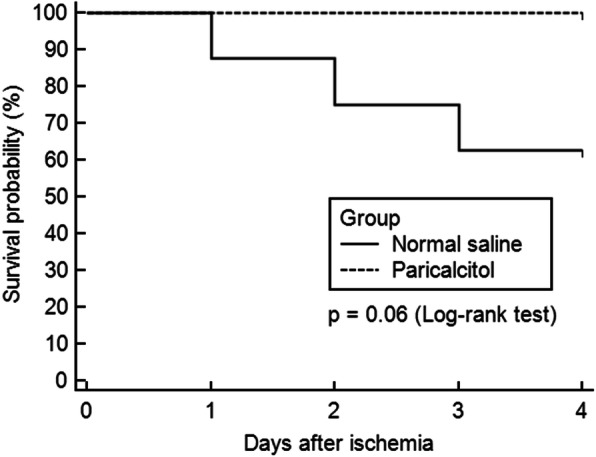
Impact of paricalcitol on survival. Paricalcitol administration did not significantly improve 96-h survival (*P* = 0.06). However, all rats in the paricalcitol group survived 96 h after cerebral ischemia

The 96-h survival rate was 100% in the paricalcitol group and 62.5% in the control group. Seizure activity was observed in the rats that did not survive. However, the survival rate was not significantly different between the groups according to the log-rank test (*P* = 0.06) (Fig. 3). Survival duration, daily NFS, and seizure activity are described in the supplemental table.

### Motor coordination

**Fig. 4:**
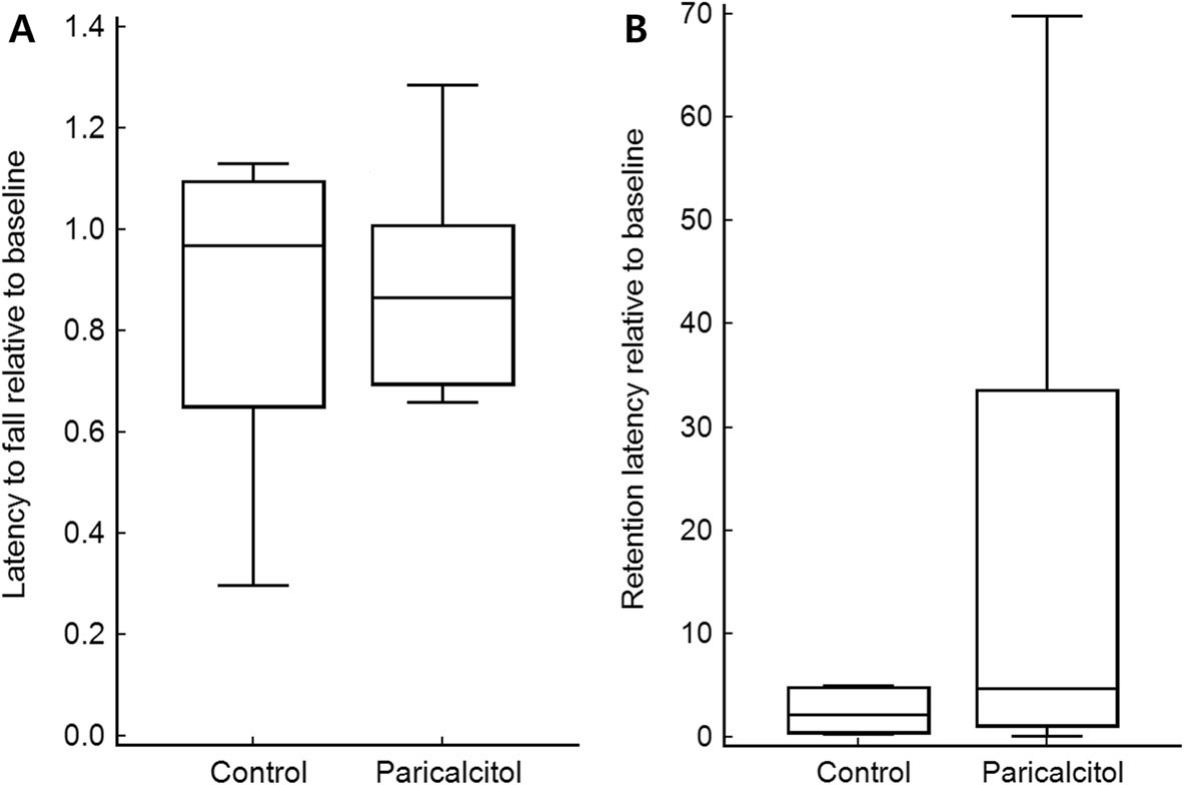
Impact of paricalcitol on motor function (**a**) and memory function (**b**). Paricalcitol administration did not improve motor coordination after cerebral ischemia (*P* = 0.56) (**a**). Although five rats in the paricalcitol group never entered the escape chamber, the median retention latency relative to baseline was not significantly different between the groups (*P* = 0.38) (**b**). The box-and-whisker plot represents medians and interquartile ranges

Three days after ischemia, the median latency to fall from a rotating rod relative to baseline in the control group was 0.97 (IQR, 0.65 to 1.09), compared with 0.87 (IQR, 0.69 to 1) in the paricalcitol group. Paricalcitol treatment did not significantly improve motor coordination (*P* = 0.56) (Fig. 4a).

### Memory function

Four days after ischemia, median retention latency relative to baseline in the control group was 2.18 (IQR, 0.51 to 4.76), compared with 4.75 (IQR, 1.17 to 33.5) in the paricalcitol group. Although retention latency was not statistically different between the groups (*P* = 0.38), five paricalcitol-treated rats never entered the escape compartment. By contrast, all of the rats in the control group entered the escape compartment (Fig. 4b).

### Neuronal degeneration

**Fig. 5:**
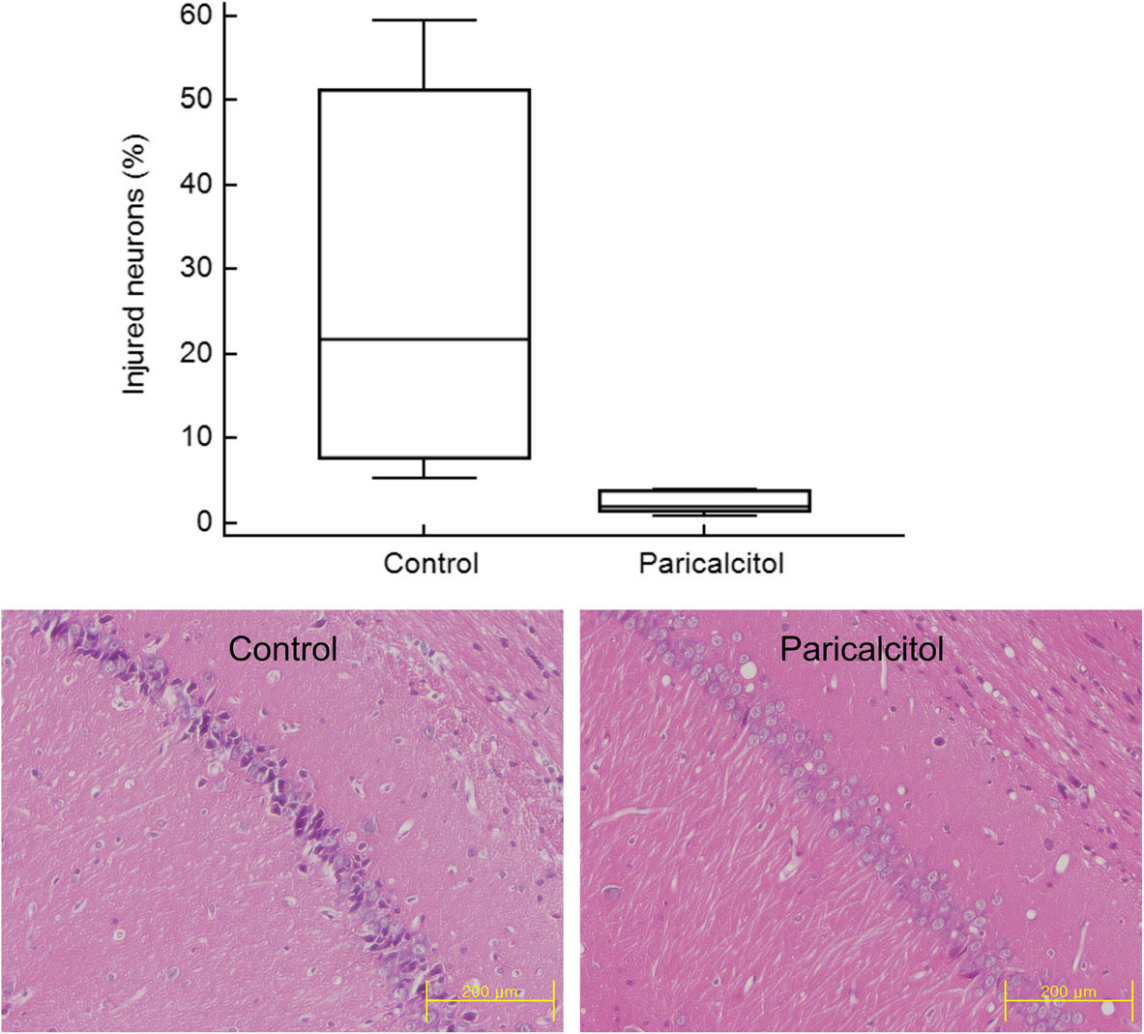
Hematoxylin-eosin stain, original magnification × 200. Neuronal degeneration of hippocampal Cornu Ammonis 1 regions 4 days after cerebral ischemia. Paricalcitol administration significantly attenuated neuronal injury (*P* = 0.01)

Four days after ischemia, the median percentage of injured neurons in the control group was 21.88% (IQR, 7.74 to 51.26), compared with 2.04% (IQR, 1.48 to 3.79) in the paricalcitol group. Paricalcitol treatment significantly attenuated neuronal degeneration in the CA1 region of the hippocampus compared with control saline treatment (*P* = 0.01) (Fig. 5).

## Discussion

This study revealed that paricalcitol has neuroprotective properties after global cerebral ischemia. The experimental rat model of global cerebral ischemia does not result in long-lasting focal neurological deficits [21]. Therefore, rats that survive longer than 1 day after four-vessel occlusion recovered well, showing equivalent NFSs between the groups. Global HIBI is caused by various clinical conditions, such as asphyxia, profound shock, and cardiac arrest. Among those conditions, cardiac arrest results in the most devastating injury. In a rat model of cardiac arrest, neurologic deficits last longer than in the four-vessel occlusion model [20]. Further studies are needed to identify the efficacy of paricalcitol in improving long-term neurological outcomes after severe HIBI such as that caused by cardiac arrest. Nevertheless, the significant improvement in NFS 2-h post-ischemia in the paricalcitol group seen in this study indicated that paricalcitol contributed to short-term recovery of neurologic function. Paricalcitol administration significantly attenuated neuronal injury. Although there was no significant difference in the passive avoidance test, all rats in the control group entered the dark chamber while only 37.5% of the rats in the paricalcitol group entered the dark chamber, which suggests a trend toward preservation of memory function in the paricalcitol group. The sample size in the present study may not have been adequate to evaluate the effects of paricalcitol on survival or cognitive and motor function; it was chosen to identify differences in neuronal injury.

Previous studies demonstrated that vitamin D is neuroprotective in a rat model of transient focal cerebral ischemia [11, 12]. Furthermore, several studies have shown that vitamin D, vitamin D3, and calcitriol supplementation improve recovery from traumatic brain injury [22–26]. The proposed mechanism of vitamin D neuroprotection is mediated via anti-inflammatory and anti-apoptotic effects [6–8, 27]. Vitamin D also acts as an antioxidant and promotes axonal regeneration [28–30]. As paricalcitol is a vitamin D receptor agonist that diffuses through the blood-brain barrier and shows relatively few side effects such as hypercalcemia and hyperphosphatemia, it may be a more appropriate neuroprotective drug than vitamin D itself for clinical applications [30, 31]. Until now, paricalcitol has mainly been used for treatment of hyperparathyroidism associated with chronic kidney disease. Despite its limited use in the management of neurological disorders, a study demonstrated that paricalcitol has antiepileptic properties mediated by antioxidant activity [32].

There are some limitations to this study. The NFS system used in the current study was originally developed to evaluate outcomes in the cardiac arrest model [20]. This scoring system may be inappropriate for evaluation of delayed hippocampal injury. In addition, the results of the passive avoidance test were compromised by the decreased activity of the injured rats. Therefore, memory function was not significantly different between the groups by this measure. We did not identify the optimal neuroprotective dose of paricalcitol. A high dose of paricalcitol may result in complications such as hypercalcemia and hyperphosphatemia. However, we did not measure calcium and phosphate levels. The optimal paricalcitol dosing regimen, which would have substantial neuroprotective efficacy with minimal adverse effects, remains unknown. In addition, this study did not investigate intracellular signaling pathways. Thus, the neuroprotective mechanism of paricalcitol was not evaluated. Perhaps, the neuroprotective effects of vitamin D are identical to those of paricalcitol. However, paricalcitol is a selective vitamin D receptor activator, with distinct and varying levels of non-selective vitamin D receptor activation [31]. Additional studies are required to elucidate the mechanisms underlying the neuroprotective effects of paricalcitol.

## Conclusions

Paricalcitol administration attenuated neuronal injury after transient global cerebral ischemia. It remains to be determined if paricalcitol improves survival and neurological outcomes in a clinically relevant model such as a cardiac arrest model. Overall, paricalcitol should be considered as a potential neuroprotective drug, and further study would be warranted.

## Supplementary information

**Additional file 1.** Supplemental table. Rat’s daily status during follow-up

## Data Availability

The datasets used and/or analyzed during the current study are available from the corresponding author upon reasonable request.

## Abbreviations

HIBI: Hypoxic-ischemic brain injury
IACUC: Institutional animal care and use committee
NFS: Neurological function score
IP: Intraperitoneal
CA: Cornu Ammonis
IQR: Interquartile range
HE: Hematoxylin-eosin
